# Developing a mesophilic co-culture for direct conversion of cellulose to butanol in consolidated bioprocess

**DOI:** 10.1186/s13068-015-0266-3

**Published:** 2015-06-12

**Authors:** Zhenyu Wang, Guangli Cao, Ju Zheng, Defeng Fu, Jinzhu Song, Junzheng Zhang, Lei Zhao, Qian Yang

**Affiliations:** School of Life Science and Technology, Harbin Institute of Technology, Harbin, 150001 China; State Key Laboratory of Urban Water Resource and Environment, Harbin Institute of Technology, Harbin, 150090 China; School of Food Science and Engineering, Harbin Institute of Technology, Harbin, 150090 China

**Keywords:** Cellulose, Co-culture, Biobutanol, Conversion, Consolidated bioprocessing

## Abstract

**Background:**

Consolidated bioprocessing (CBP) of butanol production from cellulosic biomass is a promising strategy for cost saving compared to other processes featuring dedicated cellulase production. CBP requires microbial strains capable of hydrolyzing biomass with enzymes produced on its own with high rate and high conversion and simultaneously produce a desired product at high yield. However, current reported butanol-producing candidates are unable to utilize cellulose as a sole carbon source and energy source. Consequently, developing a co-culture system using different microorganisms by taking advantage of their specific metabolic capacities to produce butanol directly from cellulose in consolidated bioprocess is of great interest.

**Results:**

This study was mainly undertaken to find complementary organisms to the butanol producer that allow simultaneous saccharification and fermentation of cellulose to butanol in their co-culture under mesophilic condition. Accordingly, a highly efficient and stable consortium N3 on cellulose degradation was first developed by multiple subcultures. Subsequently, the functional microorganisms with 16S rRNA sequences identical to the denaturing gradient gel electrophoresis (DGGE) profile were isolated from consortium N3. The isolate *Clostridium celevecrescens* N3-2 exhibited higher cellulose-degrading capability was thus chosen as the partner strain for butanol production with *Clostridium acetobutylicum* ATCC824. Meanwhile, the established stable consortium N3 was also investigated to produce butanol by co-culturing with *C. acetobutylicum* ATCC824. Butanol was produced from cellulose when *C. acetobutylicum* ATCC824 was co-cultured with either consortium N3 or *C. celevecrescens* N3-2. Co-culturing *C. acetobutylicum* ATCC824 with the stable consortium N3 resulted in a relatively higher butanol concentration, 3.73 g/L, and higher production yield, 0.145 g/g of glucose equivalent.

**Conclusions:**

The newly isolated microbial consortium N3 and strain *C. celevecrescens* N3-2 displayed effective degradation of cellulose and produced considerable amounts of butanol when they were co-cultured with *C. acetobutylicum* ATCC824. This is the first report of application of co-culture to produce butanol directly from cellulose under mesophilic condition. Our results indicated that co-culture of mesophilic cellulolytic microbe and butanol-producing clostridia provides a technically feasible and more simplified way for producing butanol directly from cellulose.

## Introduction

Considering the fluctuating prices of gasoline and the shortage of fossil fuel reserves, to develop a renewable and cost-efficient biofuel becomes a pressing mission. Butanol, as a renewable energy carrier, has aroused more and more attention in the last decades. Compared with traditional biofuel ethanol, butanol has many advantages such as lower vapor pressure, higher energy content, non-hygroscopic, and can be completely miscible with gasoline or diesel in any ratios [1–3]. Among various butanol production processes, anaerobic butanol production from cellulosic biomass received great attention owing to its environmental and social sustainability benefits [4, 5]. Current strategies to produce butanol from this feedstock usually involve in an enzymatic hydrolysis step to convert cellulosic biomass into monosaccharides before anaerobic fermentation [6–9]. Although many efforts have been made to improve cellulase enzymes, it is still a major contributor to the total cost of cellulosic biofuel production [10, 11].

An alternative process that aims to reduce production costs is the consolidated bioprocessing (CBP), in which cellulase production, cellulose hydrolysis, and fermentation are completed in one step [12, 13]. CBP is therefore considered as the most economically attractive approach for converting cellulosic biomass into biobutanol. Despite several *Clostridium* spp., including *Clostridium acetobutylicum*, *Clostridium beijerinckii*, and *Clostridium saccharoperbutylacetonicum*, have been reported to produce butanol from various substrates such as agricultural residues [14] and wastewater algae [15], none of them can directly convert cellulose into butanol.

Co-cultures have been widely studied to address the limitations in substrate utilization by individual strains for the production of various bioproducts. For example, it was reported that the co-culture of *Bacillus* sp. SGP1 and *Clostridium tyrobutyricum* ATCC 25755 to produce butyric acid from sucrose [16]. Geng et al*.* discussed the effect of key factors on hydrogen production from cellulose in a co-culture of cellulolytic bacterium *Clostridium thermocellum* DSM 1237 and a non-cellulolytic hydrogen-producing bacterium *Clostridium thermopalmarium* DSM 5974 [17]. Apparently, co-culture of different microorganisms by taking advantage of their specific metabolic capacities provides a promising method to improve the substrate conversion and the product yield. Recently, the co-culture of a thermophilic cellulolytic strain *C. thermocellum* JN4 and a butanol-producing strain *C. saccharoperbutylacetonicum* N1-4 was investigated to produce butanol directly from cellulose [18]. Although considerable amount of butanol from cellulose was observed in this co-culture system, one of the problems is the different optimum temperatures required for the saccharification and fermentation stages. Cellulase production and saccharification with thermophilic bacterium is best done around 60 °C, while butanol fermentation usually occurs in the mesophilic condition, at about 37 °C. Moreover, the butanol-producing microorganisms, such as *C. saccharoperbutylacetonicum* and *C. acetobutylicum*, have been reported to even have no growth when the culture temperature exceeded 42 °C [2, 9, 19]. The mismatched temperatures for cellulose hydrolysis and butanol fermentation obviously influence the efficiency of butanol production from cellulose. Accordingly, the application of mesophilic cellulolytic microbe in co-culture system for butanol production from cellulose is of great interest. In this way, the process is more advantageous in practical applications due to being more economically feasible and less energy intensive compared with using thermophilic cellulolytic microbe as a partner in co-culture system.

In this study, an enriched stable consortium N3 and a purified strain *Clostridium celevecrescens* N3-2, which exhibited high activity of cellulose degradation at 35 °C, were firstly obtained by multiple subcultures from cellulose medium. To demonstrate the application of isolated cellulolytic cultures, we next performed co-culture fermentations with *C. acetobutylicum* ATCC824 for butanol production with filter paper as carbon source. This work is expected to provide useful information for assessing the feasibility of using mesophilic cellulolytic microbe in co-culture with butanol-producing clostridia for direct butanol production from cellulose.

## Materials and methods

### Enrichment cultures and isolation

Environmental samples, used for enrichment anaerobic cellulose degradation microflora, were collected from wheat straw compost, soil beneath wheat straw compost, rumen fluid, rumen solids, fresh cattle dung, rooted wood crumb, cattle dung compost, soil beneath cattle dung compost, and the activated sludge from wastewater digestion reactor. These samples were suspended in sterilized oxygen-free water with the ratio of solid and liquid 1:10 (*w*/*v*) and fiercely oscillated for 1 h followed by a static settlement for 10 min. Then, 10 mL supernatants were added into 90 mL enrichment medium, which contained (per liter) 10.0 g of filter paper, 1.0 g of (NH_4_)_2_SO_4_, 1.0 g of NaCl, 3.0 g of K_2_HPO_4_, 1.5 g of KH_2_PO_4_, 0.2 g of MgSO_4_, 0.5 g of CaCO_3_, 0.5 g of cysteines, and 0.2 mL trace elements solution [20]. The pH value was adjusted to 6.5 with 5 M NaOH. Nitrogen (99.9 %) was used to form the anaerobic conditions. After 6-day fermentation with shaking (100 rpm) at 37 °C in an IS-RDH1 incubator shaker (Crastal Technology (Shenzhen) Co., Ltd.), 10 mL liquid cultures were then transferred to the brand-new enrichment medium for the second generation subculture. This process was repeated multiple times until the culture had a unique microbial community which was indicated by PCR-denaturing gradient gel electrophoresis (DGGE) analysis (see below).

To obtain cellulose-degrading isolates, the stable microbial consortium was diluted and plated on solid medium containing 10 g/L of microcrystalline cellulose (Avicel, PH101) instead of filter paper as sole carbon source, while the other components were the same with enrichment medium. Colonies surrounded by clear zones were subcultured to cellulose agar plates. Replicate plating was done several times to ensure the purity of the isolated colonies. Isolates with high butanol production potential from cellulose were identified and tested.

### DGGE

Total genomic DNA was extracted from enriched microbial consortium N3 using Bacteria DNA Mini Kit (Watson Biotechnologies, Inc., China) according to the manufacturer’s instructions. DNA extracts was used as a template for amplification of the 16S rRNA V3 fragment using Ex-Taq DNA polymerase (TAKARA Biotechnology Co., Ltd.). Primers used in this amplification process were BSF338/352 (5′-actcacccgtccgcca-3′) and BSR534/518 (5′-attaccgcggctgctgg-3′). The reactions were performed in a Peltier thermal cycler (Bio-Rad Laboratories, Inc.) with the conditions of initial denaturation at 94 °C for 5 min, followed by 35 cycles of denaturation at 94 °C for 45 s, annealing at 60 °C for 45 s and extension at 72 °C for 1 min, and ended by a final extension at 72 °C for 10 min. The amplified 16S rRNA V3 fragments were analyzed by DGGE using the DCodet™ system (Bio-Rad) with a gradient concentration of denaturing agent ranging from 30 to 60 % in polyacrylamide gel. The DNA fragments recovered from the gel were used as templates for re-amplification under the reaction conditions described above, and the resulting PCR products were cloned for sequencing.

### 16S rRNA gene sequencing

The isolate genome DNA was extracted and used as templates for PCR amplification with primes BSF8/27 (5′-agagtttgatcctggctcag-3′) and BSR1492/1474 (5′-ggttaccttgttacgactt-3′). Amplification was performed in a 9700 PCR meter (Bio-Rad Laboratories, Hercules, USA) with the conditions as mentioned by Chen [21]. The sequences were initially compared to the available databases using the BLASTn sever to determine their approximate phylogeny [22]. A phylogenetic tree was constructed using the neighbor-joining method provided in MEGA version 5.1 [23].

### Batch fermentation tests

Fermentation studies were conducted in oxygen-free medium which contained the same components with enrichment medium except for 30 g/L filter paper cellulose. In the co-culture system, a volume of 2 mL microbial consortium N3 or isolate *C. celevecrescens* N3-2 in their exponential-growth phase was added into 96 mL fermentation medium, after 48 h fermentation, another volume of 2 mL *C. acetobutylicum* ATCC824, a butanol-producing strain purchased from China General Micro-biological Culture Collection Center (CGMCC), also in its exponential-growth phase, was added in.

In all cases, the fermentation was carried out at 37 °C and 100 rpm, with a final reaction volume of 100 mL. During fermentation, samples were taken at predetermined intervals. To check data reproducibility, triplicate sets were carried out in each experiment.

### Analytical methods

Liquid products of fermentation (ethanol, acetone, butanol, acetate, and butyrate) were analyzed by using gas chromatography (GC; 6890N, Agilent Technologies, Santa Clara, CA), and gases were measured by a thermal conductivity detector after separation by GC (GCSC2) (Shanghai Analytical Apparatus, Shanghai, China) as described by our previous study [24]. Sugar concentration during fermentation was determined using a high-performance liquid chromatography (HPLC) system (LC-10A, Shimadzu Corp., Kyoto, Japan) following the method reported by Cao [25]. Cell mass was determined indirectly by measuring the total protein [26]. Briefly, the fermentation broth was firstly disintegrated by ultrasonication, then the supernatant was collected after centrifugation at 12,000× *g* for 5 min and tested with Coomassie brilliant blue (CBB) by spectrophotometric analysis at 595 nm according to the method of Bradford. For residue cellulose analysis, the fermentation broths containing cell mass and cellulose were centrifuged at 12,000× *g* for 15 min to separate the supernatant and precipitate. Then, the precipitate was determined gravimetrically after drying at 80 °C for 2 days with non-inoculated medium as a control. After that, the residue cellulose was calculated by subtracting the amount of cell mass which was determined indirectly by measuring the total protein. The activities of endoglucanase, exoglucanase, and β-glucosidase were determined using the supernatant of the fermentation broth after a centrifugation at 12,000× *g*. The substrates for each enzyme were CMC-Na, microcrystalline cellulose, and salicin, respectively. The reactions were carried out at the temperature of 55 °C in pH 6.0 for 30 min according to the method reported by Cao [11]. Then, the reducing sugar was measured and calculated into enzyme activities. One unit of enzyme activity (IU) was defined as the amount of enzyme which produced 1 μmol of reducing sugar per 1 min. For carbon balance calculations, the elemental composition of the microbes was assumed to be C_5_H_7_NO_2_ [27]. Electron balances were calculated a ratio of oxidized products to reduced products (O/R ratio) as a function of the available electrons per mole of the substrate and products [28, 29]. To check data reproducibility, triplicate sets were carried out in each experiment.

## Results

### Enrichment of high bioactivity of cellulolytic microbial consortium

**Fig. 1:**
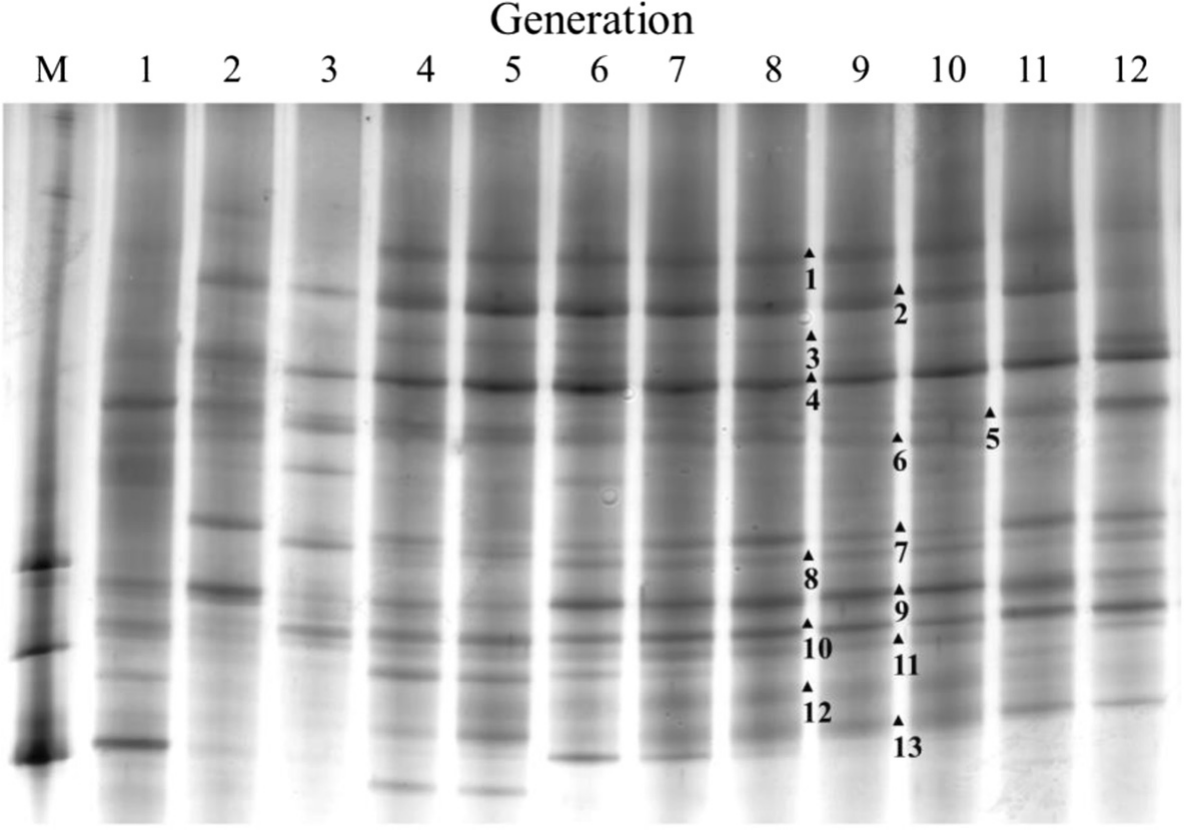
Composite microbes and stabilities of microbial consortium N3 by denaturing gradient gel electrophoresis (DGGE). Patterns coded by *number* were sequenced for further phylogenetic analysis

For obtaining highly efficient cellulolytic microbial consortium, medium with filter paper as substrate was separately inoculated with nine kinds of inocula mentioned in the”Enrichment cultures and isolation” subsection. After incubation at 35 °C for 6 days under anaerobic condition, numerous cultures displayed decomposition of filter paper. To further establish stable microbial consortium with high cellulose-degrading ability, continuous sub-cultivations were run in the filter paper medium. After several cycles, a consortium yielded from fresh cattle dung, designated N3, exhibited superior performance over other consortia with the cellulose degradation more than 60 % (Table 1). Consistent with apparent weight loss of cellulose, the cellulase activities including endoglucanase, exoglucanase, and β-glucosidase reached the highest with the levels of 0.67 ± 0.05, 0.54 ± 0.10, and 0.25 ± 0.04 U/mL, respectively, for consortium N3 among tested cultures. In the light of the robust anaerobic growth and efficiency of cellulose degradation, the consortium N3 was therefore investigated for its microbial community. Based on the 16S rRNA gene-targeted PCR-DGGE profiles for each sub-culturing generation, the microbial community structure of N3 tended to be stable after eight consecutive sub-cultivations. It can be inferred from Fig. 1 that some of the bands appeared in the starting generations disappeared at later period, indicating that the natural elimination occurred from the artificial inheriting generation to generation. The microbial diversity of stable consortium was analyzed. The major bands were cut and purified for sequencing. After BLAST analysis, bands 1, 2, 3, 5, 7, and 12 were identified as *Proteiniphilum acetatigenes*, *Clostridium ramosum*, *Clostridium celerecrescens*, *Desulfovibrio africanus*, uncultured *Ethanoligenens* sp., and uncultured *Clostridium* sp., bands 6 and 8 were identified as *Aminobacterium colombiense*, and bands 4, 9–11, and 13 were identified as uncultured bacterium (Table 2), respectively. All of these microorganisms were assumed to be involved in the degradation of filter paper, and their co-existence in the stable consortium N3 suggests that they live together in a commensal relationship and interact with each other to contribute to cellulose degradation.

**Table 1:** Cellulose degradation and cellulase activities for different microbial consortiums

	CK^a^	Consortium N3	Strain N3-1^b^	Strain N3-2^b^	Strain N3-3^b^	Strain N3-4^b^	Strain N3-5^b^	Combination of isolated strains^c^	Pooled SEM
Degradation (%)	4.76 f	63.35 a	7.08 e	29.83 b	11.57 d	18.36 c	12.33 d	21.77 c	1.63
Endoglucanase activity (U/mL)	0 f	0.67 a	0.13 cd	0.26 b	0.11 de	0.08 e	0.19 bc	0.22 bc	0.04
Exoglucanase activity (U/mL)	0 d	0.54 a	0.08 c	0.16 b	0.02 d	0.03 d	0.09 c	0.17 b	0.05
β-Glucosidase activity(U/mL)	0 d	0.25 a	0.12 b	0.23 a	0.06 c	0.10 b	0.14 b	0.13 b	0.02

For each row of the table, values with the different lowercase letters are significantly different (*P* < 0.05) while values with the same letter mean no significant difference (*P* > 0.05)

^a^CK means a control without any inoculum

^b^Strains N3-1, N3-2, N3-3, N3-4, and N3-5 stand for five different isolations screened from consortium N3, corresponding to *Proteiniphilum acetatigenes*, *C. celerecrescens*, *C. saccharolyticum*, *C. ramosum*, and *Clostridium* sp., respectively

^c^Combination of isolated strains was a recombination of the isolated strains from N3-1 to N3-5 in the same ratio as an artificial consortium

**Table 2:** Retrieve results of DGGE bands by BLASTn and sequence match for different culture samples

Bands	Most similar sequence (accession number)	Identity (%)
1	*Proteiniphilum acetatigenes* strain TB107 (NR_043154.1)	99
2	*Clostridium ramosum* strain JCM1298 (AB595128.1)	99
3	*Clostridium celerecrescens* strain 18A (NR_026100.1)	99
4	Uncultured bacterium clone B94 (JX843695.1)	99
5	*Desulfovibrio africanus* strain DSM 2603 (NR_026351.1)	98
6, 8	*Aminobacterium colombiense* strain DSM 12261 (NR_074624.1)	97
7	Uncultured *Ethanoligenens* sp. clone E6_27w (JX099826.1)	97
9	Uncultured bacterium clone BD15476 (JQ191120.1)	93
10, 11, 13	Uncultured bacterium clone FL_ 1aaa02c10 (EU775022.1)	95
12	Uncultured *Clostridium* sp. clone F8_27w (JX099828.1)	98

### Functional bacteria isolation and characterization

Serial dilutions of stabilized consortium N3 culture were plated on solid cellulose (Avicel PH101) media, enabling selection of colonies surrounded by clear zones. In total, five representative colonies with extensive clearing zones were screened. Subsequently, the capability of degrading cellulose was tested for isolated strains. As shown in Table 1, all isolates showed substantial amounts of degradation of filter paper. However, the degradation efficiency of cellulose for these isolated strains was all inferior to the stable consortium N3, this is in agreement with previous studies that natural microflora exhibits higher conversion rate on cellulosic biomass than the use of pure cultures [30, 31]. To confirm the cellulolytic activities of isolated strains, carboxymethyl cellulose (CMC), Avicel, and cellobiose were used as substrates for extracellular enzymes assay. Although all isolates showed lower endoglucanase, exoglucanase (avicelase), and cellubiose activities than the consortium N3, strain N3-2 isolated from consortium N3 displayed the most effective on cellulose degradation among isolated strains even higher than the combination of isolated five strains at the same ratio with each other. The high activities of cellulase reported here for strain N3-2 was obtained under non-optimized culture condition. This value is as much as the cellulase activities reported for the other well-known mesophilic anaerobic cellulolytic strains [32, 33]. It is therefore of interest to determine whether strain N3-2 was clustered with the dominant DGGE banding sequences. Based on the similarity analysis of the 16S rRNA gene sequence, strain N3-2 had the highest identity of 99.5 % with *C. celevecrescens*, indicating that the dominated functional bacterium presented in the stable enrichment culture was successfully isolated.

### Co-culture with *C. acetobutylicum* ATCC 824

**Fig. 2:**
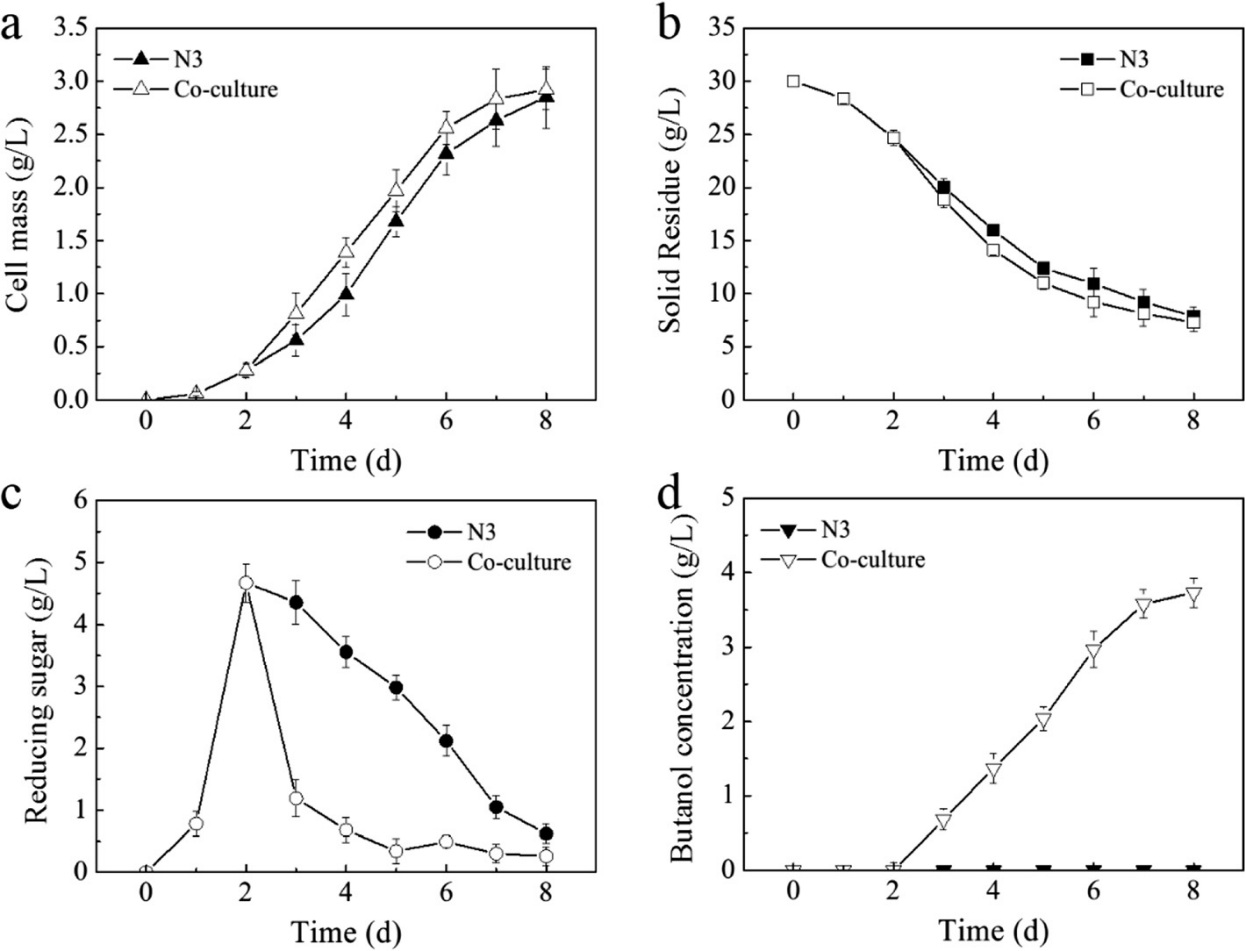
Time course of growth, substrate consumption, sugar accumulation, and butanol formation by consortium N3 and its co-culture with *C. acetobutylicum* ATCC 824 (**a–d**)

**Fig. 3:**
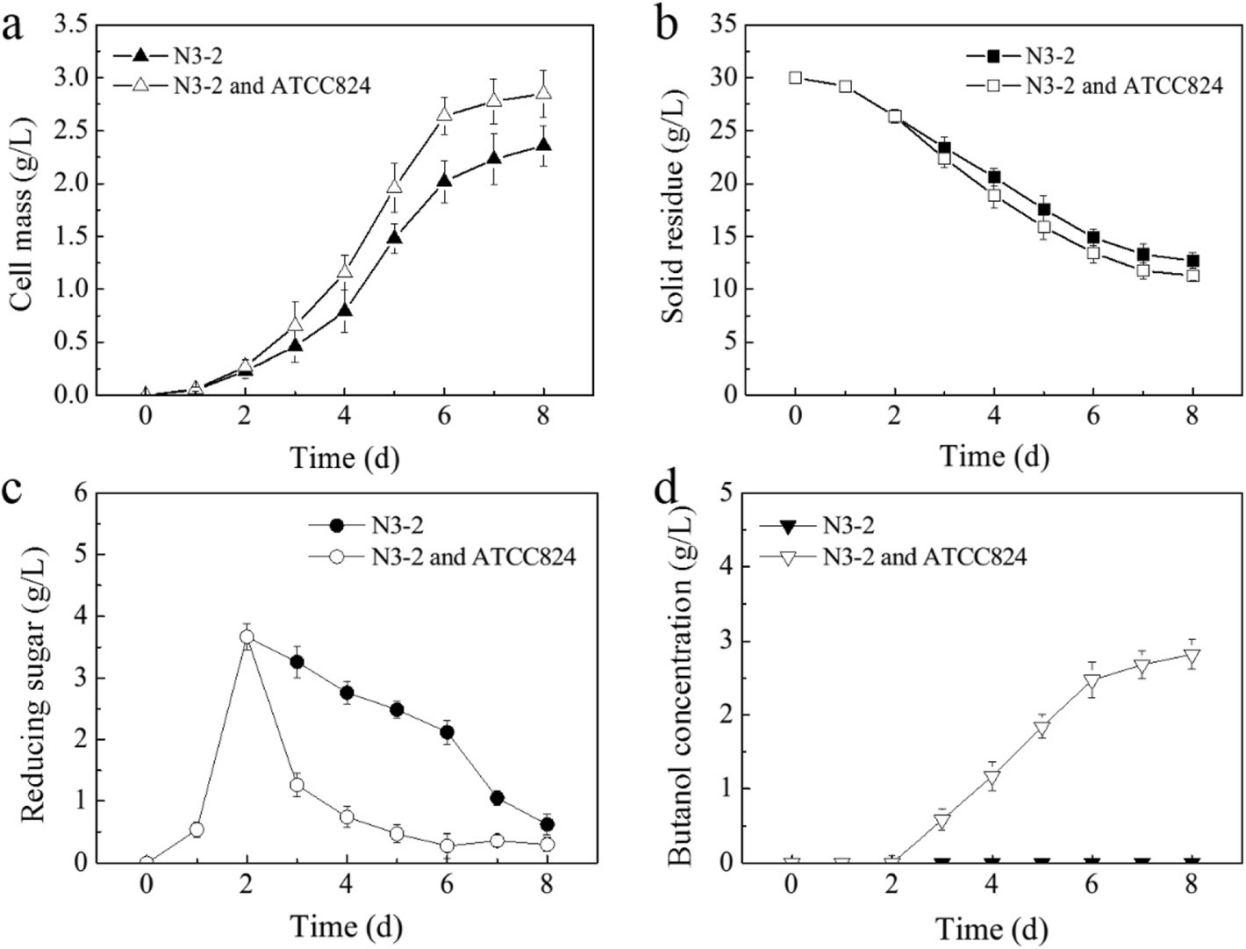
Time course of growth, substrate consumption, sugar accumulation, and butanol formation by *C. celevecrescens* N3-2 and its co-culture with *C. acetobutylicum* ATCC 824 (**a–d**)

Since butanol-producing clostridia can hardly utilize cellulose as carbon source, a hydrolysis step before fermentation is necessary to convert cellulose into reducing sugars. So a co-culture system with cellulolytic bacteria obtained in this study was designed here, permitting butanol-producing microorganism to ferment cellulose to butanol. To evaluate this, the stable consortium N3 and the purified strain *C. celevecrescens* N3-2 were co-cultured with *C. acetobutylicum* ATCC 824, respectively. In the meanwhile, growth of stable consortium N3 or *C. celevecrescens* N3-2 by themselves on cellulose medium was used as control. As shown in Fig. 2, the behavior of co-culture consortium N3 and *C. acetobutylicum* ATCC 824 appeared to be similar to that of the monoculture consortium N3, regarding cell growth and cellulose consumption. Nevertheless, the reducing sugar accumulation and butanol formation between co-culture and monoculture had significant difference. In the case of co-culture, the reducing sugar accumulated in the first 48-h fermentation sharply decreased after *C. acetobutylicum* ATCC 824 was added into the broth. While it showed a placid decrease in the monoculture of consortium N3. Little or no butanol was observed in the monoculture of consortium N3. However, when *C. acetobutylicum* ATCC 824 was co-cultivated with, a significant amount of butanol as high as 3.73 g/L was produced after 8 days of fermentation, indicating that consortium N3 could produce butanol from cellulose only when it was co-cultured with *C. acetobutylicum* ATCC 824. The same observations go for the *C. celevecrescens* N3-2 in its co-culture with *C. acetobutylicum* ATCC 824 even though the amount of butanol formed by the latter co-culture was lower than that formed by the former consortium co-culture (Fig. 3). This may be caused by lower cellulolytic activity of *C. celevecrescens* N3-2 than consortium N3, which in turn resulted in low productivity of butanol.

### Carbon balances

In order to understand and compare the capabilities of co-culture and monoculture for cellulose degradation and butanol production, the carbon and redox balances in the fermentation process were determined in the cellulose medium containing 3 % filter paper. Consortium N3, *C. celevecrescens* N3-2, co-culture of consortium N3 + *C. acetobutylicum* ATCC 824 and *C. celevecrescens* N3-2 + *C. acetobutylicum* ATCC 824 grown to the stationary phase without pH control revealed carbon recoveries up to 96.9 % and O/R ratio close to 1 (Table 3). The distribution of carbon among all major components showed that microbial consortium N3 alone had higher levels of ethanol, acetic acid, and propionic acid and *C. celevecrescens* N3-2 alone had higher amount of ethanol, propionic acid, and butyric acid than those in co-cultures of N3 + ATCC 824 and N3-2 + ATCC 824, respectively, while significant amount of butanol and acetone were observed in co-cultures, indicating that the carbon flow of co-culture was significant different from monoculture. It is therefore speculated that the established co-cultures favored butanol production. Based on the consumption of cellulose, the butanol yield reached 0.145 ± 0.008 g/g of glucose equivalent and 0.134 ± 0.010 g/g of glucose equivalent for the co-cultures of N3 + ATCC 824 and N3-2 + ATCC 824, respectively. No significant difference of butanol yield was observed for these two co-cultures even though lower concentration of butanol was obtained in co-culture of N3-2 + ATCC 824 (Fig. 3), which further indicated that lower butanol production in N3-2 + ATCC 824 was mainly caused by lower cellulolytic activity than N3.

**Table 3:** Carbon and redox balances of the fermentations in different conditions

Cultures	Carbon consumption (mM C)	Ethanol (mM C)	Acetate (mM C)	Acetone (mM C)	Propionic acid (mM C)	Butanol (mM C)	Butyrate (mM C)	Biomass (mM C)	CO_2_ (mM C)	Percentage of carbon in butanol (%)	H_2_ (mM)	Carbon balance (%)	O/R ratio
A^a^	799.6	209.6	119.0	0	36.6	0	74.5	126.1	178.5	0	74.2	93.1	0.98
B^a^	840.3	39.3	80.5	86.3	0	201.6	88.3	129.2	189.4	24.0	139.9	96.9	1.10
C^b^	640.7	74.3	56.4	0	42.1	0	189.2	104.4	132.7	0	70.7	93.5	0.98
D^b^	692.6	50.8	70.4	73.3	0	152.4	63.1	108.0	143.2	22.0	131.5	95.5	1.03

^a^Conditions A and B stand for fermentation using microbial consortium N3 alone and co-culture of N3 and ATCC 824

^b^Conditions C and D stand for fermentation using strain N3-2 alone and co-culture of N3-2 and ATCC 824

## Discussion

CBP of cellulosic feedstock for butanol production using mesophiles offers additional advantages in processing over the use of thermophilic microbial systems in terms of potential savings on capital and operating costs and matched saccharification and fermentation culture conditions. In order to find complementary organisms to the butanol producer that allows simultaneous saccharification and fermentation of cellulose in consolidated bioprocess to butanol in their co-culture, a highly efficient and stable consortium N3 and an isolate *C. celevecrescens* N3-2, capable of growing at 35 °C from cellulose, were obtained and incubated in the co-culture system with *C. acetobutylicum* ATCC 824. Neither consortium N3 nor *C. celevecrescens* N3-2 produced butanol from cellulose degradation in the monocultures, but when they were co-cultured with a butanol-producing bacterium *C. acetobutylicum* ATCC 824, significant amount of butanol were achieved in the co-cultures. This should be attributed to the synergetic metabolic activities of both co-cultured species. The cellulolytic consortium N3 and *C. celevecrescens* N3-2 can hydrolyze cellulose to glucose but cannot utilize glucose to produce butanol, while *C. acetobutylicum* ATCC 824 can utilize glucose efficiently to produce solvents. Additionally, the function of the consortium N3 or the isolate N3-2 might to be more than just generating sugars for the *C. acetobutylicum*, the accumulation of acetate and butyrate for consortium N3 and isolate N3-2 maybe have a boosting effect on butanol production in the co-culture system since these acids are known precursors to butanol [34]. This study clearly demonstrated the advantage of applying a co-culture in cellulose fermentation under mesophilic condition to improve the butanol production which provides a technically feasible and more simplified way for producing butanol directly from cellulose while a full economic analysis is warranted in the future.

Although the attractive aspects of butanol concentration and productivity were not observed by the co-culture system in this study when they were compared with processes (separated hydrolysis and fermentation (SHF) and simultaneous saccharification and fermentation (SSF)) featuring exogenous cellulase addition and the co-culture of using thermophilic cellulolytic *C. thermocellum* ATCC 27405 and butanol-producing strain *C. saccharoperbutylacetonicum* N1-4 (Table 4), the butanol production reported here was obtained with the cellulose-degrading microbe and butanol-producing strain grown on non-optimized medium under non-optimized cultivation conditions. It should be noted that the concentration of butanol in co-cultures of N3 + ATCC 824 and N3-2 + ATCC 824 was substantially higher than those obtained in co-cultures of thermophilic *C. thermocellum* ATCC 27405 + *C. acetobutylicum* ATCC 824 and *C. thermocellum* ATCC 27405 + *C. beijerinckii* NCIMB 8052 (Table 4), indicating that co-culture with different butanol-producing strains also had a significant influence on the effectiveness of butanol production. This may be caused by the genes which are responsible for producing solvent induced to a different extent under different co-culture systems [18]. Optimization of co-culture conditions such as pH, temperature, mixing ratio and identification of the ecological relationship between the organisms, and targeted genetic engineering of the organisms for improving the concentration and rate of butanol production from cellulose should be addressed carefully in the future. Overall, this is the first report of application of co-culture to produce butanol directly from cellulose under mesophilic condition. Our results indicated that co-culture of mesophilic cellulolytic microbe and butanol-producing clostridia provides a technically feasible and more simplified way for producing butanol directly from cellulose.

**Table 4:** Comparison of butanol production in different fermentation processes using cellulosic substrates

Microorganism	Fermentation process	Substrate	Butanol concentration (g/L)	Butanol yield (g/g substrate)	Butanol productivity (g/L h)	Reference
Consortium N3 + *C. acetobutylicum* ATCC 824	Co-culture	Filter paper	3.73	0.145	0.020	This study
*C. celevecrescens* N3-2 + *C. acetobutylicum* ATCC 824	Co-culture	Filter paper	2.69	0.134	0.014	This study
*C. thermocellum + C. saccharoperbutylacetonicum* N1-4	Co-culture	Crystalline cellulose	7.90	0.198	0.037	[18]
*C. thermocellum + C. beijerinckii* NCIMB 8052	Co-culture	Crystalline cellulose	2.05	–	–	[18]
*C. thermocellum + C. acetobutylicum* ATCC 824	Co-culture	Crystalline cellulose	<1.0^a^	–	–	[18]
*C. beijerinckii* BA101	SHF	Corn fiber	6.40	0.138	0.073	[35]
*C. beijerinckii* NCIMB 8052	SHF	Corncob residue	5.60	0.130	0.057	[6]
*C. saccharoperbutylacetonicum* N1-4	SHF	Sago starch	10.40	0.29	0.072	[36]
*C. acetobutylicum* MTCC 481	SHF	Rice straw	2.10	1.04	0.017	[37]
*C. acetobutylicum* ATCC 824	SSF	Wheat straw	5.05	0.127	0.084	[24]
*C. beijerinckii* P260	SSF	Wheat straw	7.40	0.113	0.164	[20]
*C. acetobutylicum* ATCC 824	SSF	Seepweed	3.50	0.101	0.101	[38]

En dash means not determined

^a^Butanol concentration as indicated in reference [18]

## Conclusions

This study investigated the feasibility of using newly screened consortium N3 and pure strain *C. celevecrescens* N3-2 to co-culture with *C. acetobutylicum* ATCC 824 to produce butanol directly from cellulose. Results showed that significant amount of butanol as high as 3.73 g/L was achieved by co-culture consortium N3 and *C. acetobutylicum* ATCC 824. Considerable butanol yield of 2.69 g/L was also acquired by co-culture *C. celevecrescens* N3-2 and *C. acetobutylicum* ATCC 824. This is the first study to employ cellulolytic mesophiles to co-culture with butanol-producing strain to produce butanol directly from cellulose. Overall, the results obtained in this study suggest that co-culture butanol-producing clostridia with mesophilic cellulolytic microbe could be a technically feasible and more simplified way for producing butanol from cellulose in consolidated bioprocess.

## Abbreviations

BLAST: Basic local alignment search tool
CBP: Consolidated bioprocessing
CGMCC: China General Micro-biological Culture Collection Center
DGGE: Denaturing gradient gel electrophoresis
GC: Gas chromatograph
HPLC: High-performance liquid chromatography
PCR: Polymerase chain reaction
SHF: Separate hydrolysis and fermentation
SSF: Simultaneous saccharification and fermentation
